# Genome editing demonstrates that the −5 kb Nanog enhancer regulates Nanog expression by modulating RNAPII initiation and/or recruitment

**DOI:** 10.1074/jbc.RA120.015152

**Published:** 2020-12-20

**Authors:** Puja Agrawal, Steven Blinka, Kirthi Pulakanti, Michael H. Reimer, Cary Stelloh, Alison E. Meyer, Sridhar Rao

**Affiliations:** 1 Department of Cell Biology, Neurobiology, and Anatomy, Medical College of Wisconsin, Milwaukee, Wisconsin, USA; 2 Blood Research Institute, Versiti, Milwaukee, Wisconsin, USA; 3 Department of Pediatrics, Medical College of Wisconsin, Milwaukee, Wisconsin, USA

**Keywords:** embryonic stem cells, transcription regulation, gene expression, gene regulation, pluripotency, super-enhancers, enhancers, Nanog, 4OHT, 4-hydroxytamoxifen, CRE, *cis* regulatory element, ESC, embryonic stem cells, gRNA, guide RNA, HDR, homology directed repair, LIF, leukemia inhibitory factor, NOS, Nanog, Oct4, and Sox2, RNAPII, RNA Polymerase II, SE, super-enhancer, TBST, tris buffered saline with tween, TES, transcriptional end site, TF, transcription factors, TSS, transcriptional start site

## Abstract

Transcriptional enhancers have been defined by their ability to operate independent of distance and orientation in plasmid-based reporter assays of gene expression. At present, histone marks are used to identify and define enhancers but do not consider the endogenous role of an enhancer in the context of native chromatin. We employed a combination of genomic editing, single cell analyses, and sequencing approaches to investigate a *Nanog*-associated *cis*-regulatory element, which has been reported by others to be either an alternative promoter or a super-enhancer. We first demonstrate both distance and orientation independence in native chromatin, eliminating the issues raised with plasmid-based approaches. We next demonstrate that the dominant super-enhancer modulates *Nanog* globally and operates by recruiting and/or initiating RNA Polymerase II. Our studies have important implications to how transcriptional enhancers are defined and how they regulate gene expression.

Gene expression is regulated by two types of genetic elements: *Trans* elements typically encode proteins such as transcription factors (TFs), which subsequently bind *cis*-regulatory elements (CREs) that must be on the same DNA molecule as the gene they regulate. Different types of CREs have historically been classified based upon their behavior in plasmid-based reporter assays (1, 2). For almost 40 years it has been accepted that promoters are required to be in the correct orientation and immediately adjacent to the gene they regulate, whereas enhancers operate independent of both distance and orientation. The advent of enhancer-specific epigenetic signatures based on histone marks such as H3K27Ac or H3K4me1 permit genome-wide identification of enhancers, which then demonstrate enhancer activity in reporter assays (3, 4). However, plasmid-based assays are limited for multiple reasons. First, they do not fully recapitulate native chromatin structure, and therefore represent a highly artificial system. Second, they typically are performed on smaller (<500 bp) DNA sequences rather than on the larger chromatin domains of many highly active enhancers. Third, they cannot precisely link a given enhancer sequence to the gene(s) they may regulate *in vivo*. As such, plasmid assays are far more effective at confirming DNA sequences with enhancer potential, rather than definitively identifying them as enhancers.

The advent of sequencing-based chromosomal conformation capture techniques has allowed the measurement of genome-wide enhancer-gene interactions, potentially identifying enhancer:gene regulatory interactions (5, 6). Of interest, this approach demonstrates that many enhancers interact with multiple genes and vice versa but in isolation are insufficient to properly determine if an enhancer is required for gene(s) expression (7, 8). The classic approach to address this question is through genetics, namely, deleting a putative enhancer and measuring the mRNA levels of nearby genes, a method made highly feasible through genomic editing approaches such as CRISPR-Cas9. One important point is that, although these approaches can identify which gene(s) are regulated by an enhancer, many of the mechanistic details of how the enhancer regulates transcription to modulate gene expression are not elucidated through solely this approach.

Multiple models of enhancer-mediated gene expression exist within the literature. Early theories postulated that enhancers looped in to interact with promoters and recruited RNA Polymerase II (RNAPII) to the gene’s promoter (9). More recently, multiple mechanisms that focus on enhancers regulating transcriptional elongation have been proposed, including promoter-proximal pause release of RNAPII through various mechanisms (reviewed in Chen *et al.* 2018). It has also been proposed that enhancers modulate transcriptional bursting, or the periods of time during which transcription is active, which represents a combination of initiation and elongation (11, 12). New studies demonstrate that among enhancers there is a subclass of highly active enhancers called “super-enhancers” (SEs, (13, 14)), which may potentially form phase-separated droplets within the nucleus to concentrate transcriptional machinery around highly transcribed genes (15). It is important to note this current model remains to be definitively established. Collectively, this literature indicates that there may be multiple mechanisms by which enhancers regulate gene expression.

The extended *Nanog* locus is a unique locus to study how super-enhancers regulate gene expression and pluripotency. The *Nanog* locus (150 kb) contains a number of different pluripotency-associated genes including *Dppa3*, *Gdf3*, and *Apobec1* (7, 16). It also contains three SEs (−5, −45, and +60, based upon distance in kilobases from *Nanog* TSS) that interact with *Nanog* and behave as enhancers in reporter assays (7). Recent work from our group and others has demonstrated that these three enhancers are super-enhancers based upon several criteria, including high levels of the epigenetic mark H3K27Ac, robust binding by the Mediator complex, and production of enhancer-transcribed RNAs (13, 14). One group has argued that the −5 SE/CRE is actually an alternative promoter, emphasizing that plasmid-based approaches are insufficient to determine if a DNA element is a promoter or enhancer (17). In this study we demonstrate that the −5 CRE is an enhancer by confirming that it operates in a distance- and orientation-independent fashion through genomic approaches and regulates *Nanog* by modulating RNAPII initiation or recruitment.

## Results

### The −5 *Nanog* CRE is required for embryonic stem cell pluripotency in a Nanog-dependent manner

**Figure 1:**
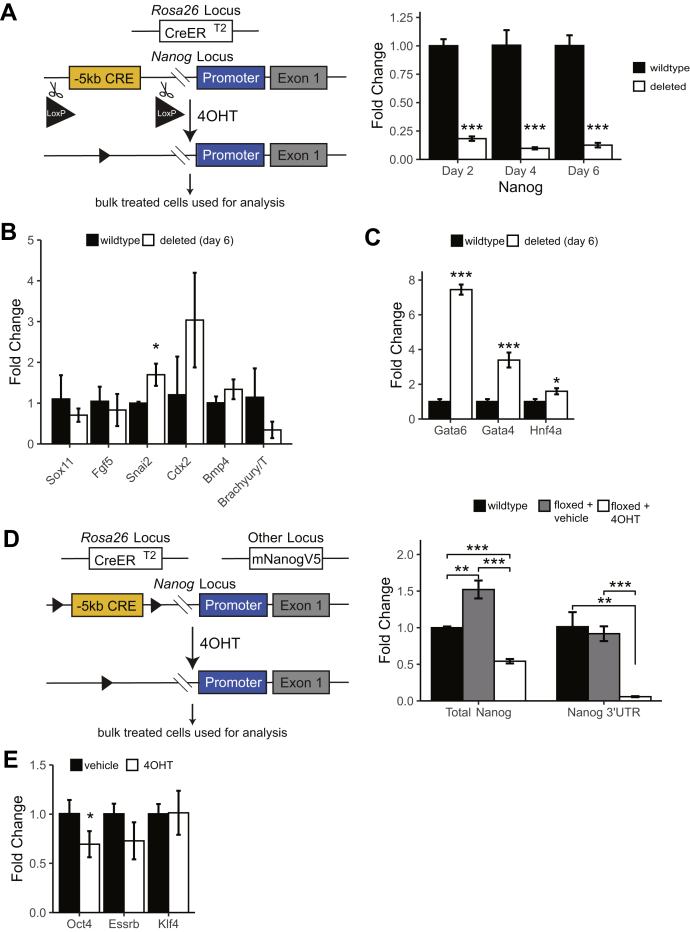
**Deletion of the −5 CRE.***A*, biallelic deletion of −5 CRE was achieved by inserting two loxP sites around the enhancer and cells were treated with tamoxifen (4OHT) for 6 days. *Left panel*, schematic; *right panel*, Nanog mRNA levels in bulk vehicle or 4OHT-treated cells. n = 3. *B*, day 6 mRNA expression of mesodermal, ectodermal, and trophectodermal differentiation markers. n = 3. *C*, day 6 mRNA expression of endodermal promoting transcription factors. n = 3. *D*, stable biallelic deletion of the −5 CRE was achieved by rescuing with a mouse Nanog cDNA. Endogenous Nanog expression is measured *via* the Nanog 3’UTR, while Total Nanog measured endogenous and the exogenous expression. *Left panel*, schematic; *right panel*, mRNA levels in bulk treated cells. n = 3. *E*, pluripotency markers in bulk treated cells in −5 CRE-deleted cells expressing Nanog in *trans* shown in (*D*). n = 3. All mRNA levels measured by RT-qPCR and shown as 2^∧^ΔΔCT compared with wildtype or vehicle treated. ∗*p* < 0.05, ∗∗*p* < 0.01, ∗∗∗*p* < 0.001 Student’s two sample *t* test. CRE, *cis*-regulatory element.

Previously, we demonstrated that the −5 CRE physically interacts with the *Nanog* promoter by chromosomal conformation capture and could also activate *Nanog* expression in plasmid-based reporter assays (7). Monoallelic deletion of the −5 CRE causes a 50% reduction in *Nanog* expression (7). However, we were unable to recover mouse embryonic stem cells (ESCs) that exhibited biallelic deletion of the −5 CRE, leading us to hypothesize it is required for pluripotency. We refer to this element as a CRE rather than an SE because one group has previously reported that this element is an alternative promoter (17). To identify if this element is required for *Nanog* expression, we used genomic editing to insert a tamoxifen (4OHT)-inducible Cre-recombinase (CreER^T2^) into the constitutively expressed *Rosa26* locus in ESCs to facilitate conditional deletions and then biallelically inserted *loxP* sites to flank a 2.5-kb region of the −5 CRE to encompass two Nanog, Oct4, and Sox2 (NOS) binding sites (Fig. S1*A*, Fig 1*A*, left). Insertion of loxP sites causes no change to *Nanog* expression (Fig. S1, *B*–*C*). Treatment with 4OHT induces complete biallelic deletion of the −5 CRE as compared with vehicle treated (ethanol; Fig. S1*D*, Fig. 1*A*, left). ESCs began to differentiate and became nonadherent, consistent with a loss of pluripotency, following 4OHT exposure. Staining for the pluripotency marker alkaline phosphatase was reduced in bulk cells treated with 6 days of 4OHT (Fig. S1*E* i–ii) compared with control (Fig. S1*E* iii–iv). Deletion of the −5 CRE resulted in a rapid loss of Nanog mRNA (Fig. 1*A*, right) and protein (Fig. S1*F*). By contrast, *Gdf3*, a nearby gene, showed little change in expression following deletion of the −5 CRE (Fig. S1*G*). There was also a decrease in other pluripotency-associated TFs such as *Oct4*, *Esrrb*, and *Klf4*, demonstrating a progressive collapse of the transcriptional network regulating pluripotency (Fig. S1, *H*–*J*). Consistent with previous studies showing that Nanog represses endoderm specification, RT-qPCR for key differentiation genes (Fig. 1*B*) and endoderm-promoting TFs such as *Gata4*, *Gata6*, and *Hnf4a* (Fig. 1*C*) demonstrated an increased expression in specifically the endoderm-promoting genes following 4OHT treatment. These results demonstrate that the −5 CRE is required for ESC pluripotency, likely by regulating *Nanog* expression.

Next, we hypothesized that the −5 CRE maintains pluripotency solely by regulating *Nanog* expression rather than the expression of another gene on chromosome 6 (chr6). To test this, we made a stable cell line expressing murine *Nanog* with a ubiquitous promoter (CAG; Fig. 1*D*, left). Of importance, *Nanog*^*+/−*^ animals are viable and ESCs remain pluripotent (18, 19), indicating that 50% levels of Nanog do not compromise pluripotency in mice. Endogenous *Nanog* gene expression can be followed with RT-qPCR primers amplifying the *Nanog* 3’ UTR, which is absent from the *mNanogV5* transgene. Prior to 4OHT treatment, bulk cells express *Nanog* mRNA levels approximately 50% higher than wildtype ESCs, which then falls following 4OHT treatment to 50% below wildtype (Fig. 1*D*, right). After 4OHT treatment, these cells show a profound (>90%) reduction in endogenous *Nanog* expression (Fig. 1*D*, right) and a small decrease in *Oct4* levels but no other significant change in other core pluripotency TFs such as *Esrrb* or *Klf4* (Fig. 1*E*). In addition, following 6 days of 4OHT treatment cells remain alkaline phosphatase positive (Fig. S2*A*), consistent with the *Nanog* transgene rescuing the loss of pluripotency seen following deletion of the −5 CRE (Fig. 1).

Following 6 days of 4OHT treatment on the −5 CRE floxed cells expressing individual clones were selected, expanded, and analyzed (Fig. S2*B*). These cells remain pluripotent even after 6 days of leukemia inhibitory factor (LIF) withdrawal (Fig. S2*C*, right) because of the constitutive expression of *Nanog* off the ubiquitous CAG promoter of the transgene. Western blot analysis of total Nanog in isolated individual clones shows a decrease in Nanog expression of approximately 50% upon stable deletion of the −5 CRE (Fig. S2*D*). These data demonstrate that the loss of pluripotency in cells without the *Nanog* transgene is attributable to the loss of *Nanog* expression. It should be noted that pluripotency was determined by a combination of gene expression (Fig. 1*E* and see RNA-Seq below), morphology, and alkaline phosphatase staining (Fig. S2, *A* and *C*), but we were unable to perform the most rigorous test of either teratoma formation or tetraploid complementation owing to the presence of the Nanog transgene in these cells, which prevents differentiation.

To determine if the activity of other super-enhancers within the extended *Nanog* locus changed upon deletion of the −5 CRE, we measured enhancer-transcribed RNA levels, which are widely used as a measure of enhancer activity (7, 13, 20). In individually expanded clones of the −5 CRE deletion with Nanog *in trans*, we observed a global decrease in enhancer activity (Fig. S2*E*), which may be due to the presence of the exogenous *Nanog* transgene at a decreased level (50%). However, we cannot rule out that the decreased enhancer activity is a direct result of −5 CRE deletion. From here forward, all experiments using a biallelically deleted −5 CRE deletion were done using clonal cell line(s) that supply *Nanog* in *trans* to prevent a loss of pluripotency.

**Figure 2:**
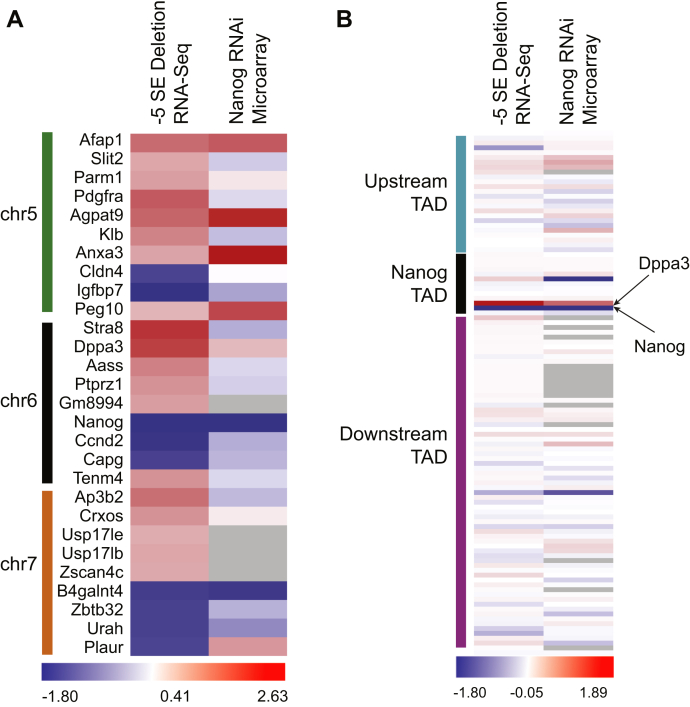
**The −5 CRE regulates Nanog exclusively.***A*, differentially expressed genes, defined to be at least 2-fold, statistically significant (adj *p* < 0.05, n = 3) change, on chromosome 5,6, and 7 *via* RNA-Seq. Nanog RNAi microarray data are shown for comparison (20). *B*, differentially expressed genes, irrespective of significance or fold-change, on chromosome 6 within the Nanog TAD, one TAD upstream and one downstream. The −5 SE-deleted cells express Nanog in *trans* to prevent a loss of pluripotency. CRE, *cis*-regulatory element; SE, super-enhancer; TAD, topologically associated domain.

To determine if the −5 CRE solely regulates *Nano*g we used RNA-Seq to identify other altered transcripts. We used the cell line described in Figure 1*E*, a stable line with the −5 CRE deleted that expresses exogenous mNanogV5 and compared it with the floxed −5 CRE expressing exogenous mNanogV5 cell line. First, we identified genes on chr6, which showed at least a 2-fold, statistically significant change (adj *p*-value < 0.05) between samples (Fig. 2*A*). As a control, we also compared our data with previously published RNA-Seq data where *Nanog* was depleted by RNAi (21). We further queried changes on chr5&7 to estimate gene expression changes secondary to *trans* effects from changes in Nanog protein levels. We observed that gene expression changes in the −5 CRE deleted line mimics the *Nanog* RNAi data, implying that all changes are due to alteration in the levels of Nanog protein operating in *trans.* On chr6, none of the altered genes were within 1 MB of *Nanog* except *Dppa3*. The observed increase in *Dppa3* expression was expected, since it is directly repressed by Nanog protein binding to its promoter (7). These data suggest that the −5 CRE only regulates *Nanog*. To further clarify if loss of the −5 CRE affected the expression of other genes, *in cis* we queried expression changes of all genes within the *Nanog* topologically associated domain (TAD) as well as the two adjacent topologically associated domains (22), irrespective of statistical significance or fold-change (Fig. 2*B*, Table S1). With the exception of *Nanog* and *Dppa3*, most genes showed minimal gene expression changes that were comparable with the *Nanog* RNAi data, implying this was due to reduced Nanog protein operating in *trans*. We therefore conclude that the −5 CRE exclusively regulates *Nanog* expression in ESCs, with no evidence that it regulates other genes on chr6 in *cis*. Collectively, these experiments demonstrate that the *Nanog* −5 CRE is required for pluripotency through its direct regulation of *Nanog* expression.

### The *Nanog* −5 CRE operates in a distance- and orientation-independent fashion

**Figure 3:**
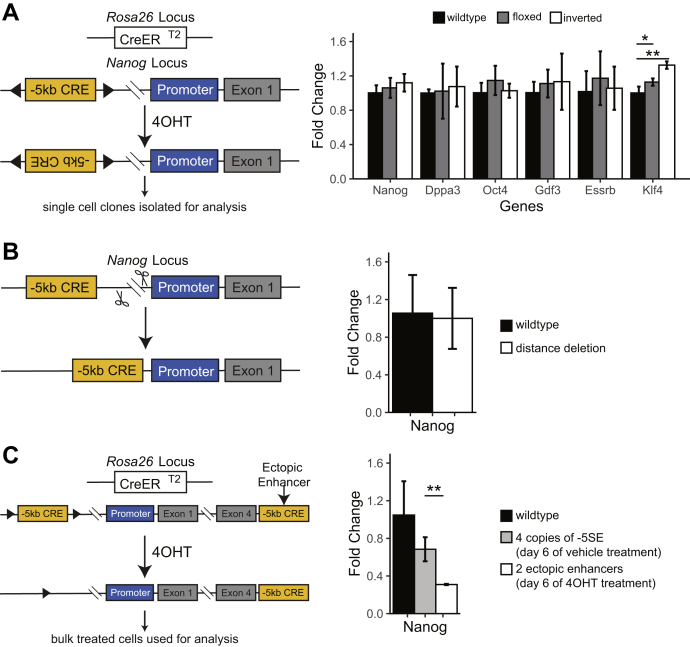
**Manipulation of the −5 CRE.***A*, the −5 CRE was flipped by inserting opposing loxP sites followed by tamoxifen (4OHT) treatment. *Left panel*, schematic; *right panel*, mRNA levels of Nanog and relevant pluripotency genes in individual isolated clones. n = 3. *B*, an approximately 2-kb region between the −5 CRE and Nanog TSS was deleted. *Left panel*, schematic; *right panel*, Nanog mRNA levels. n = 5. *C*, two copies of the −5 CRE were inserted downstream of Nanog in cells where the endogenous enhancer is floxed. Endogenous enhancers were deleted *via* treatment with tamoxifen. *Left panel*, schematic; *right panel*, mRNA levels of Nanog in bulk cells treated with vehicle or 4OHT. n = 3. All mRNA levels measured by RT-qPCR and shown as 2^∧^ΔΔCT compared with wildtype. ∗*p* < 0.05, ∗∗*p* < 0.01 Student’s two sample *t* test. None of the cell lines shown express Nanog in *trans*. CRE, *cis*-regulatory element.

The *Nanog* −5 CRE in plasmid assays acts independent of distance and orientation and has been extensively referred to as an enhancer within the literature (23, 24). By contrast, in at least one report the −5 CRE was considered an alternative promoter that played a critical role in regulating pluripotency through an alternative *Nanog* isoform (17). Given this ambiguity we chose to definitively establish if this element had enhancer activity within the context of normal chromatin with our 4OHT-inducible Cre-LoxP system by inserting one of the *LoxP* sites in the opposite orientation (Fig. 3*A*, left). In this configuration, Cre activation by 4OHT treatment will induce biallelic “flipping” of the −5 CRE continuously between the two orientations. Following treatment with 4OHT, individual clones were isolated and expanded and biallelic inversion of the −5 CRE was verified by PCR (Fig. S3*A*). On comparison of the biallelic inversion with the wild-type orientation of the −5 CRE, we observed no statistically significant changes in the expression of *Nanog*, *Dppa3*, *Oct4*, or *Esrrb* and only minor changes in *Klf4* (Fig. 3*A*, right). To verify there was no change in the Nanog protein banding pattern to indicate a potential change in protein isoforms, we performed Western blots and did not observe any changes in the banding pattern (Fig. S3*B*). These data are consistent with the −5 CRE regulating *Nanog* expression in an orientation-independent manner, a classic property of an enhancer but not a promoter. Although we cannot rule out that this CRE can simultaneously act as both an enhancer and an alternative promoter, our data demonstrate that any alternative isoforms produced *via* the −5 CRE as a promoter (17) are dispensable for pluripotency.

We next hypothesized that the −5 CRE would also operate independent of distance from the *Nanog* transcriptional start site (TSS). To determine this, we first deleted the intervening ≈2 kb between the −5 CRE and promoter (Fig. S3*C*, Fig. 3*B*, left) and found no significant change in *Nanog* expression (Fig. 3*B*, right). Next, we biallelically inserted an additional copy of the −5 CRE between the *Nanog* transcriptional end site (TES) and the nearest CCCTC binding factor site to ensure it remained within the same insulated neighborhood (Fig. S3, *D*–*E*, Fig. 3*C*, left, 25). Insertion of the additional −5 CRE caused no change in *Nanog* mRNA levels (Fig. 3*C*, right). Treatment with 4OHT for 6 days resulted in deletion of the endogenous −5 CRE in bulk cells and caused a reduction in *Nanog* mRNA by approximately 50% (Fig. 3*C*, right). Expression of other key pluripotency markers such as *Oct4*, *Esrrb*, and *Klf4* were unchanged, indicating that pluripotency was maintained (data not shown). The partial recapitulation of *Nanog* expression and pluripotency by the ectopic enhancers is consistent with the −5 CRE operating independent of distance, albeit less effectively than its native chromatin position (Fig. 3*C*, right). *Nanog* expression in the cells only containing the ectopic enhancer is ≈35% compared with wildtype, which is higher than the ≈10% *Nanog* seen on the conditional deletion of the −5 CRE in bulk cells (Fig. 1*A*) and near 0% *Nanog* we see on true complete deletion in individual clones (Fig. 1*D*, right). We do observe an insignificant decrease in *Nanog* in cells with ectopic and endogenous enhancers, treated with vehicle; however, this could be due to minor changes in chromatin architecture from the insertion of the ectopic enhancers. One reasonable explanation for the reduced *Nanog* expression with the ectopic enhancer is that the −5 CRE includes a larger chromatin domain, whereas we inserted only the core ≈2.5 kb containing two NOS sites into an alternative location. This may imply that additional sequences surrounding the core are required for full activity. Alternatively, moving the enhancer further away may prevent it from fully activating Nanog. Nonetheless, this demonstration of enhancer function represents a highly feasible, native chromatin approach to confirm that a DNA element is an enhancer. Collectively, these data demonstrate that the −5 CRE is an enhancer, and we will hereafter refer to it as the −5 SE (14).

### Constituent enhancers within the −5 SE are additive in regulating *Nanog* expression

**Figure 4:**
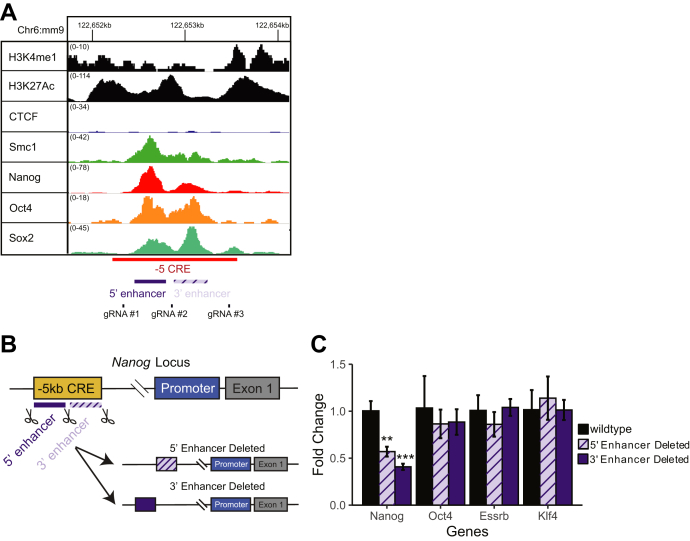
**−5 SE constituent enhancers are both required.***A*, Integrated Genome Viewer snapshot of the −5 SE showing two constituent enhancers and locations of guide RNAs. The *x*-axis is genomic position; *y*-axis is normalized tag count. *B*, schematic. *C*, mRNA levels of Nanog and relevant pluripotency genes when two constituent enhancers within the −5 SE were deleted. n = 3. All mRNA levels measured by RT-qPCR and shown as 2^∧^ΔΔCT compared with wildtype. ∗∗*p* < 0.01, ∗∗∗*p* < 0.001 Student’s two sample *t* test. SE, super-enhancer.

Several groups have demonstrated that, within a super-enhancer, a single, smaller constituent enhancer is required for proper SE function, with the remaining constituent enhancers being dispensable for regulating gene expression (26). To determine if the −5 SE has a dominant constituent enhancer, we first reviewed published chromatin immunoprecipitation coupled with next-generation sequencing (ChIP-seq) datasets from other groups to determine if there were constituent enhancers within the larger −5 SE. We observed that there were two distinct regions occupied by the classic pluripotency transcription factors Nanog, Oct4, and Sox2 (NOS, Fig. 4*A*). To determine if one or both constituent enhancers were critical to pluripotency, we deleted each individually with CRISPR-Cas9 using a pair of distinct guide RNAs (gRNAs) (Fig. 4, *A*–*B*, Fig. S3*F*). It is surprising that we were able to recover biallelically deleted clones of the individual 5’ or 3’ constituent enhancers without difficulty. Deletion of either the 5’ or 3’ constituent enhancer results in an approximately 50% reduction in *Nanog* mRNA, but these reductions were insufficient to alter pluripotency as measured by *Oct4*, *Esrrb*, or *Klf4* expression (Fig. 4*C*). This demonstrates that neither the 5’ nor the 3’ constituent enhancer is required for pluripotency, even though together they promote normal *Nanog* expression. Thus, the two constituent enhancers function in an additive fashion and are required for proper *Nanog* expression.

### The −5 SE regulates expression in all cells

Previous studies from our laboratory demonstrated that monoallelic deletion of the −5 SE, biallelic deletion of the −45 SE, and biallelic deletion of the +60 SE have different effects on *Nanog* expression despite each enhancer physically interacting with the gene, as shown by chromosomal conformation capture (7). Specifically, deletion of the −45 SE causes a 50% decrease in *Nanog* expression, whereas deletion of the +60 SE had no change in *Nanog* expression. Work from this study has further shown that the −5 SE is critical to *Nanog* expression, as there is a 90% decrease in *Nanog* expression upon biallelic deletion (Fig. 1*E*, right). We hypothesized that each enhancer may operate on distinct subpopulations of cells, which we could not distinguish using a bulk population. Specifically, the −5 SE could be regulating a larger proportion of high-*Nanog*-expressing cells (Fig. S4, *A*–*C*).

**Figure 5:**
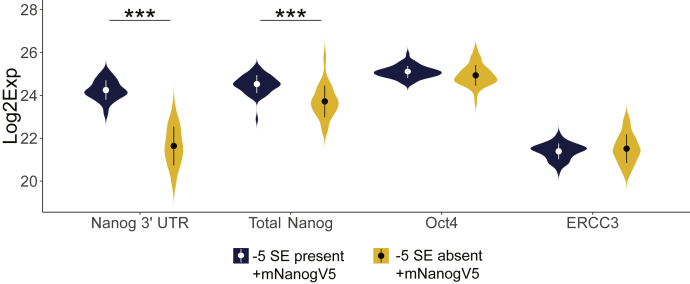
**−5 SE operates in all cells.** Single-cell RT-qPCR for endogenous *Nanog via Nanog 3’UTR*, *Total Nanog*, *Oct4*, and *ERCC3* in −5 SE-deleted cells. Expression is depicted as Log2exp relative to the Limit of Detection, as described by Fluidigm. n = 51 to 77. ∗∗∗*p* < 0.001 by Mann–Whitney test. The −5 SE-deleted cells express Nanog in *trans* to prevent a loss of pluripotency. SE, super-enhancer.

To investigate this possibility, we performed single-cell RT-qPCR on the −5 SE biallelically deleted cells with *Nanog* in *trans* (Fig. 1*E*). As described above, these are stably deleted clones that remain pluripotent in the presence of the exogenously supplied *Nanog* allowing us to examine changes in endogenous *Nanog* due to the loss of the −5 SE. If the −5 SE operates on *Nanog* in all cells, we should observe a uniform reduction in *Nanog* mRNA levels (Fig. S4*B*). By contrast, if the −5 SE regulates a different population of cells, we should observe a bimodal distribution of *Nanog* expression following enhancer deletion (Fig. S4*B*). It should be noted that, although some groups have shown that *Nanog* has bimodal expression in single cells (27), the presence of truly bimodal *Nanog* expression is debated (28, 29). Of interest, we found a uniform reduction in both total Nanog and endogenous *Nanog* expression as measured by the 3’ UTR when the −5 SE is deleted (Fig. 5). Calculation of the bimodal coefficient (30) shows that both the wildtype and deleted lines demonstrate a nonbimodal distribution as both have a coefficient that is less than 5/9 (Fig. S4*D*). It should be noted that single-cell RT-qPCR data are more sensitive than bulk qPCR and the data are represented as a Log2 of expression rather than ΔΔCT. All analyzed data points are present within Table S2. Thus, the difference in the mean of the single cell expression is a 2.6-fold reduction between the control and deleted lines, which is equivalent to an ≈ 84% decrease in expression, consistent with our bulk qPCR data (Fig. 1*E*, right). Two controls, *Oct4* and *ERCC3*, showed minimal changes in expression. Collectively, these single cell experiments support a model that the −5 SE actively regulates *Nanog* expression in all ESCs grown in serum/LIF.

### The −5 SE regulates *Nanog* by regulating transcriptional initiation/recruitment

Previous studies have argued that SEs regulate gene expression through promoter-proximal pause release (hereafter referred to as pause release) of RNAPII (31, 32). Briefly, transcription begins with the recruitment of RNAPII to the promoter, which is immediately phosphorylated on Ser 5 (Ser5P) of its C-terminal domain, resulting in bidirectional transcription around the TSS and is referred to as “paused” RNAPII because it cannot elongate further into the gene body. The “pausing” of RNAPII occurs 20 to 120 bp downstream of the TSS, which must be relieved for productive gene transcription (10). “Pause release” is mediated by phosphorylation of Ser2, releasing RNAPII to transcribe the gene body and is referred to as elongating RNAPII. To measure changes in RNAPII dynamics, we performed CUT&Tag (33) with antibodies specific to total and paused (RNAPII-Ser5P) RNAPII in WT and −5 SE deleted cells with *Nanog* supplied *in trans* (0-copy cell line, Fig. 1*E*). We chose to use the 0-copy cell line to directly compare two clonal pluripotent cell lines where changes to *Nanog* are solely due to the enhancer deletion without the confounding issue of 4OHT treatment effects on the transcriptome. We note that we cannot judge if RNAPII dynamics or transcription itself may regulate the interaction between the −5 SE and the *Nanog* promoter.

**Figure 6:**
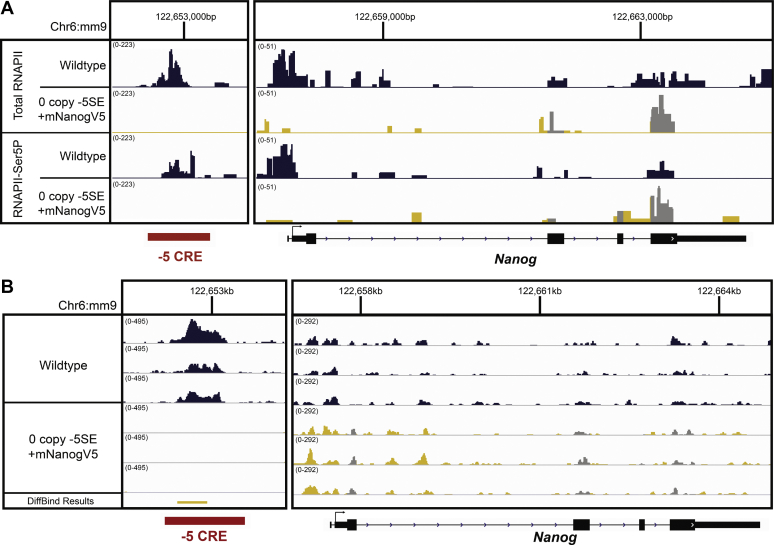
**−5 SE operates prior to RNAPII pause release.***A*, Integrated Genome Viewer snapshot of CUT&Tag for Total RNAPII (n = 1) and RNAPII-Ser5P (n = 3) in wildtype and 0 copy of −5 SE cells. The *left panel* shows the −5 SE (*y*-axis = 0–233), and the *right panel* shows Nanog (*y*-axis = 0–51). Note that the exonic regions of the 0 copy cell line are confounded by the exogenous Nanog cDNA. *B*, Integrated Genome Viewer snapshot of ATAC-Seq data, separated by each sample of each wildtype cells where the −5 SE is floxed and cells with the −5 SE completely deleted. Differential peaks identified using DiffBind are shown in the last track. The *left panel* shows the −5 SE (*y*-axis = 0–495) and the *right panel* shows Nanog (*y*-axis = 0–292). Genes and SEs are shown below. The *x*-axis is genomic position; *y*-axis is normalized read count. The −5 SE-deleted cells express Nanog in *trans* (mNanogV5) to maintain pluripotency, and overlapping regions are shown in grayscale and are not included in the analysis. SE, super-enhancer.

Depending on which phase of transcription an enhancer is regulating, RNAPII’s genomic location will change as shown in Fig. S5. As described above, RNAPII is phosphorylated on the Ser5 position of its C-terminal domain after recruitment, at which point it is paused. If recruitment is regulated by the enhancer, loss of the enhancer will cause a loss RNAPII-Ser5P at the TSS (Fig. S5-i). If pause release is being regulated, loss of an enhancer will cause a build-up of RNAPII-Ser5P that cannot be released (Fig. S5-ii). If neither of these are the steps being regulated by the enhancer, RNAPII-Ser5P enrichment will remain unchanged (Fig. S5-iii). A confounding issue of this system is that RNAPII binding at *Nanog* exons in the 0-copy cell line is obscured by the presence of the *Nanog* cDNA in *trans*, because the exogenous *Nanog* cDNA is identical to the endogenous coding regions. Therefore, we are unable to distinguish between the binding to the coding exons within *endogenous Nanog versus Nanog* cDNA supplied in *trans*. Thus, we limited our analysis to regions of the endogenous *Nanog* transcript that do not overlap with the exogenous transcript, which are the intronic and noncoding regions (blue and yellow areas in Fig. 6, *A*–*B*) and not the coding regions (grayed areas in Fig. 6, *A*–*B*). We observed a complete loss of paused RNAPII at both the −5 SE and *Nanog* in the 0-copy cell line (Fig. 6*A*), consistent with the −5 SE playing a critical role in RNAPII recruitment and/or phosphorylation on Ser5. These data indicate that the −5 SE regulates *Nanog* not through RNAPII pause-release, but rather through modulating transcriptional initiation/recruitment.

These data led us to question whether the −5 SE modulates the initial binding of RNAPII to *Nanog* by changing chromatin accessibility, rather than recruiting RNAPII. To investigate the chromatin landscape, we performed assay for transposase-accessible chromatin using sequencing (ATAC-Seq) on wildtype cells and −5 SE-deleted cells with Nanog supplied in *trans* (Fig. 1*D*). As with the CUT&Tag data, chromatin accessibility changes within the *Nanog* exons will be confounded by the presence of the exogenous mNanogV5, and thus we focused only on changes within the regions of the *Nanog* transcript that do not overlap with the mNanogV5 (intronic and noncoding regions). We observed no significant differences in chromatin accessibility within the *Nanog* locus, although there is an expected change at the −5 SE given its deletion (Fig. 6*B*). DiffBind analysis (bottom track, Fig. 6*B*) concluded that the only major change in accessibility was at the −5 SE. Thus, we conclude that the mechanism by which the −5 SE regulates *Nanog* is by modulating RNAPII recruitment/initiation but not through a change in chromatin accessibility. Although we cannot distinguish between transcriptional initiation *versus* recruitment, given the complete loss of RNAPII at *Nanog* upon deletion of the −5 SE, recruitment of RNAPII to the promoter is likely the rate-limiting step modulated by the enhancer, since generation of the initiating form of RNAPII (Ser5P) is not rate limiting. Together, these data show that the −5 SE is an enhancer critical to *Nanog* expression prior to pause-release of RNAPII.

## Discussion

Although enhancers have been well known regulators of gene expression for 40 years, it has become apparent with new technologies that they are far more numerous than classical protein-coding genes (>4-fold) and unlike promoters far more variable across tissues, implying that enhancers play a central role in regulating tissue-specific expression (34, 35, 36). Given their importance, the breadth of questions that remain within the field is profound. In particular, the reliance on plasmid-based approaches have been *de rigeur* for the formal definition of whether a *CRE* has enhancer activity. For the −5 SE, given its relative proximity to the *Nanog* TSS and the literature suggesting it may have a promoter-like activity (17), plasmid-based assays alone were unlikely to definitively address if the −5 CRE operates as an enhancer. Of importance, this DNA element may function as an alternative promoter in other contexts, such as alternative pluripotent states or in primordial germ cells, where *Nanog* is also expressed (37). Given the ease of genetically engineering ESCs using CRISPR-Cas9 technology, our initial goal was to move beyond plasmid-based approaches and utilize native cells/chromatin to determine whether the −5 SE truly displayed enhancer potential. We utilized a combination of classical genetic approaches to draw several conclusions about the −5 SE. First, the −5 SE is required for pluripotency, which can be genetically rescued by supplying Nanog *in trans*. Second, reversing the orientation of the −5 SE had no effect on pluripotency or *Nanog* expression. Third, moving the enhancer either closer to the *Nanog* TSS or within the insulated neighborhood but downstream of the TES permitted sufficient *Nanog* expression to maintain pluripotency. Of importance, these data conclusively demonstrate that the −5 SE is an enhancer. However, three caveats remain. First, although the −5 SE is an enhancer, our studies do not rule out that it can also act simultaneously as an alternative promoter, as has been suggested by others (17). Reversing the orientation of the −5 SE had no effect on *Nanog* expression and pluripotency, however, which formally demonstrates that any potential promoter activity of the −5 SE is not required for pluripotency. The second caveat is that insertion of the −5 SE downstream of the *Nanog* TES did not completely recapitulate normal expression. One reasonable explanation is that the −5 SE includes a larger chromatin domain, whereas we inserted only the core ≈2.5 kb into an alternative location. This may imply that additional sequences surrounding the core are required for full activation when it is moved into a new location. Nonetheless, this demonstration of enhancer function represents a highly feasible, native chromatin approach to assess enhancer function. Finally, given the fact that Nanog can bind to its own promoter to regulate its own expression, we cannot rule out that subtle changes in autoregulation are being disrupted through our various genetic alterations.

By the end of our studies we realized the *Nanog* locus has essentially been converted to a reporter gene, with minimal alteration beyond those described and the insertion of *LoxP* sites. This minimizes confounding variables inherent to artificial reporters such as the extensive presence of bacterial DNA sequences and/or insertion into a heterologous region of the genome. In addition, because *Nanog* is critical to ESCs, this permitted us to perform additional experiments to understand how the enhancer may modulate pluripotency and/or differentiation. One caveat is that many of our experiments were performed by supplying Nanog in *trans*, thereby permitting the cells to maintain pluripotency even when native *Nanog* expression was significantly reduced by deleting the −5 SE. Given our use of a heterologous, constitutively active promoter (CAG) and a *Nanog* cDNA that lacked the 5’ UTR, we cannot ensure that subtle changes in the temporal regulation of *Nanog* expression or Nanog protein levels were preserved. Nonetheless, supplying Nanog in *trans* was able to restore approximately heterozygous levels of *Nanog*, which is sufficient to maintain pluripotency and substantially suppress the spontaneous differentiation of ESCs (18, 19).

The recent literature has shown that there are many mechanisms by which enhancers regulate gene expression. It was previously thought that enhancers promoted the recruitment of RNAPII and other transcriptional machinery to the promoter to “promote” transcription (9). Current studies focusing on highly active enhancers have centered on their role in RNAPII pause release, which constitutes the first steps of RNAPII converting from the initiating to the elongating form (31, 38). Multiple studies have also shown that enhancers regulate transcriptional bursting (11, 12), the observation of oscillating transcriptional activity over time, in which one transcriptional burst is a period of time during which there is active transcription. Enhancers have been shown to regulate the frequency of these bursts of activity, which represent a combination of initiation and elongation. More recently, enhancers have been implicated in forming phase-separated condensates that concentrate transcriptional machinery for actively transcribed genes (15, 39, 40). Critically, these studies have not demonstrated how multiple enhancers could simultaneously regulate expression of a single gene, and if enhancers uniformly operate through the same or different mechanisms on the same gene. Since the −5 SE is indispensable for *Nanog* expression, and cannot be compensated for by another enhancer, the obvious question is whether this is because the other enhancers function through different mechanisms, or perhaps function in other pluripotent states. Deletion of each enhancer at the locus causes variable changes in *Nanog* expression (7), leading us to ask if each enhancer may play a unique role in regulating *Nanog* expression through different phases of transcription. For example, it may be that, while the −5 SE does not regulate *Nanog* through pause-release, another enhancer plays this more traditional role.

Given the broad role of enhancers in regulating tissue-specific gene expression, our work has implications for how other gene:enhancer pairs are studied. In the absence of genetic confirmation, it is difficult to confirm an enhancer:gene functional dyad based solely on plasmid-based approaches. In addition, further attention needs to be paid to the other enhancers in the region to understand how multiple enhancers work together to regulate a gene. Understanding the interplay of the three SEs around *Nanog* will further drive changes in how gene:enhancer pairs are studied, especially since they may operate through different phases of transcription to regulate expression.

## Experimental procedures

For further information and requests for reagent and resources, please contact the Lead Contact, Sridhar Rao (Sridhar.rao@versiti.org; 414-937-3841).

### Cell culture

Gelatin-adapted ESCs were utilized for all experiments. This cell line is a male, in-house generated, ICM-derived 129SVJ-derived murine ESC line, similar to the one we have used previously and cultured under similar Serum/LIF conditions (41, 42). Briefly, cells were propagated under feeder-free conditions in Dulbecco's modified Eagle's medium (Corning #10-017-CV) with the following supplements (FBS, GemBio #100-106; Penicillin/Streptomycin, Corning #30-002-Cl; MEM Nonessential Amino Acids, Corning #25-025-Cl; L-glutamine, Corning #25-005-Cl; Nucleosides, Sigma #ES-008-D; LIF, β-mercaptoethanol at the appropriate concentration). Two micromoles of 4-hydroxytamoxifen (4OHT) in 70% ethanol (EtOH) was used for all experiments and diluted 1:1000 for drug treatments with EtOH as a control.

### CRISPR-Cas9–mediated genomic editing

To generate biallelic *loxP* ESC clones, single gRNAs targeting specific regions flanking CREs were designed using the CRISPR design tool (http://crispr.mit.edu/). gRNAs were cloned into the Cas9 expressing vector px459 v2.0 (Addgene #62988, 43, 44). Single-strand DNA oligos were designed with ∼60-bp homology directed repair (HDR) arms flanking each side of the 34-bp *loxP* sequence and a restriction enzyme palindromic sequence (BamHI) for restriction digest genotyping of genomic PCR products. The *loxP* and restriction enzyme sequence was inserted between the PAM recognition sequence and the gRNA genomic targeting sequence. A single gRNA and single-strand HDR oligo were cotransfected along with the gRNA (1–2 μg of each plasmid) into 1 to 2 × 10^6^ WT ESCs using Lipofectamine 2000 (Invitrogen #1168-019) in a single well of a 6-well plate. Transfected ESCs were selected with puromycin (2 μg/ml for 2 days only) and then passaged onto 10-cm dishes at various dilutions and grown until single colonies appeared. Individual clones that were resistant to puromycin were isolated and expanded for genotyping. Primers designed outside of the HDR arms were used to genotype for enhancer deletion. Following genomic PCR, products were digested with XbaI (NEB, R0145s) for genotyping. Clones that demonstrated biallelic cutting were cloned into TOPO TA (Thermo Fisher #45-0641) for sequencing to confirm correct integration. *loxP* sequences (upstream or downstream of the targeted region) were inserted one at a time.

Cell lines described in Table S3 were generated using the following CRISPR strategy. gRNAs were cloned into px459 v2. The plasmid was digested using Bbsl (NEB, R0539) and purified. gRNA oligos were phosphorylated and annealed using T4 PNK (NEB, M0201). The cut vector and annealed oligos were ligated overnight at 16 °C. Ligated plasmids were transformed into NEB High Efficiency (NEB, C2987) bacteria, plated on LB+Amp plates and incubated overnight at 37 °C. Colonies were picked and mini-prepped for sequencing, followed by maxi-preps once gRNA presence was verified. The same transfection protocol described above was followed, and at least two single cell clones were picked and analyzed for the following cell lines.

To generate a floxed −5 *Nanog* CRE ESC line for conditional deletion the 3’ *loxP* was inserted first using a gRNA and a HDR arm (Table S4). The 5’ *loxP* sequence was subsequently inserted using a gRNA and a HDR arm. The PAM sequences adjacent to the *loxP* sequences were mutated to prevent cutting of Cas9 following HDR. The gRNAs were used to constitutively delete the −5 CRE in (7). ESCs were treated with 4OHT for 4 days at 2 μM to delete the −5 *Nanog* CRE.

To generate −5 *Nanog* CRE inverted clones we inserted the 5’ HDR oligo containing *loxP* in the opposite orientation into the ESC clone that contains the 3’ *loxP* above. The same 5’ gRNA above was used with a single-strand HDR oligo. An ESC clone with *loxP* sequences in the opposite orientations was treated with 4-OHT at 2 μM for 3 days and cells were subcloned as described above. We confirmed biallelic inversion by genomic PCR with primers inside and outside of the *loxP* sequences. Clones that demonstrated wildtype, monoallelic, and biallelic orientation were cloned into TOPO TA plasmids. At least four individual clones were then isolated and sequenced to confirm correct integration.

To insert the −5 *Nanog* CRE downstream of the Nanog gene, a single gRNA was used to stimulate HDR of a modified version of pL451 (*loxP* sequence removed). The HDR vector contains a *Neomycin resistance* (*Neo*) cassette flanked *frt* sites and by homology regions (left arm chr6:122667133–122668329, 1197 bp, mm9; right arm chr6:122668389–122669450, 1062 bp, mm9). The left arm was cloned using KpnI and SalI, and the right arm was cloned using BamHI and NotI. The enhancer (same sequence used in reporter assays in Blinka *et al.*, 2016) was inserted adjacent to the left arm using SalI and EcoRI sites. The HDR plasmid was cotransfected along with a gRNA 5’-TGGCTTGCATCCAATCTCTT-3’ chr6: 122668369, mm9 (2–3 μg of gRNA and 6 μg of HDR plasmid) into 10 x 10^6^ WT ESCs using Lipofectamine 2000. HDR vector arms and the enhancer were amplified off of a BAC (7) and fully sequenced in pBlueScript II SK(+) and matched the genomic reference sequence. Transfected ESCs were selected with puromycin (2 μg/ml first 2 days only) and G418 (350 μg/ml days 2–14) until single colonies appeared. Individual clones that were resistant to both puromycin and G418 were isolated and expanded for genotyping. *Neo* was removed by transfecting cells with a FLPe expressing plasmid driven by the CAG promoter. Primers designed outside of the HDR arms were used to genotype for enhancer insertion. Following genomic PCR to genotype, homozygous clones containing Neo were amplified and cloned into TOPO TA for sequencing to confirm correct integration.

Constituent enhancer deletions were generated using three gRNAs. Clones were genotyped using PCR primers designed around the constituent enhancers. Distance deletion clones were generated using four gRNAs. Clones were genotyped using PCR primers that surrounded the deleted portion.

All gRNAs and genotyping primers are listed in Table S4.

### Generation of murine Nanogv5 rescue cell line

The mouse Nanog sequence was synthesized by GeneArt Strings DNA Fragment (ThermoFisher). A C-terminal v5 tag was added to distinguish from endogenous Nanog protein. The synthesized DNA fragment was A-tailed and cloned into TOPO TA (Thermo Fisher #45-0641) to confirm the sequence. XhoI and NotI sites were designed at the 5’ and 3’ end of the DNA fragment so that it could be cloned into and the pPyCAG iH vector (hygromycin resistance, gifted from Austin Smith) for expression under a ubiquitous (CAG) promoter (13, 42). ESCs were electroporated with the linearized plasmid (Fsp1 NEB R0135) in the presence of hygromycin, and individual clones were isolated as we have done previously and expanded for further experiments.

### Total RNA RT-qPCR

Total RNA was harvested from cells following manufacturer’s protocol (TRIzolReagent, Invitrogen #15596018). Genomic DNA was removed from the total RNA samples using a DNA eliminator column step and passing RNA over a column following manufacturer’s protocol (RNeasy Plus Mini Kit, Qiagen#74134). Equal amounts of DNA-free total RNA were converted to cDNA using the iScript cDNA synthesis kit (Bio-Rad #1708891). Quantitative PCR (qPCR) was performed on a QuantStudio 6 Flex Real-Time PCR System (ThermoFisher). Quantifications were normalized to an internal control (Actin) for reverse transcriptase-qPCR (RT-qPCR) using the ΔΔCt method as we have done previously (7). Primers used for RT-qPCR are described in Table S4.

### Alkaline phosphatase staining

Bright-field images and alkaline phosphatase staining were performed as previously described (Rao *et al.*, 2010, Sigma 86R-1kt). Briefly, cells were plated in 10-cm dishes and treated for up to 6 days with vehicle or tamoxifen. Plates were rinsed 1x with PBS, fixed using citrate–acetone–formaldehyde fixative for 30s, and rinsed with deionized water for 45s. An alkaline-dye mixture (diazonium salt solution + deionized water + Naphthol As-BI Alkaline Solution) was added to the plate and incubated for 15 min at room temperature in the dark. The dye mixture was removed from the plates, and the plates were rinsed for 2 min with deionized water and then air dried. For LIF withdrawal experiments, 5000 cells were plated in 6-well dishes and provided new media daily with no LIF for 6 days. Cells were then stained with alkaline phosphatase as described above.

### Western blots

Proteins were extracted in radioimmunoprecipitation buffer and quantified as described in (45). A total of 10 μg of protein was loaded in each well of a gel (Bio-Rad # 567-1094, 567-1095, 456-1036). Blots were blocked in 5% milk/tris buffered saline with tween (TBST) for 20 min to 2 h at room temperature (RT) or overnight at 4 °C. Primary antibodies to Nanog (Millipore; Cat # 5731) was used at 1:1000, beta-Actin (Sigma; Cat # a5441) was used at 1:5000 in 5% milk/TBST or GAPDH-HRP (Cell Signaling Tech; 51332S) at 1:3000 in 5% milk/TBST for 90 min at RT or overnight at 4 °C. Blots were then washed with TBST and secondary antibody donkey anti-rabbit IgG-HRP (Santa Cruz; Cat # sc2313) was used at 1:5000 for 30 to 60 min at RT for Nanog. For beta-Actin, a secondary antibody (Santa Cruz; Cat # sc2064) goat anti-mouse IgM-HRP was used at 1:5000 for 30 min at RT. Blots were washed with TBST and then antibody labeled proteins were detected using Amersham ECL Prime Western Blotting Detection Reagent (Cat # RPN2232).

### RNA-Seq

RNA-Seq libraries were generated using the NEBNext Ultra RNA Library Prep Kit for Illumina (NEB #E7530). Libraries were quantified using the NEBNext Quant Kit (NEB #E7630) and Agilent Tapestation 2200 (D1000 tapes) and were sequenced on a NextSeq 500(36 × 36 PE). Library preparation and sequencing were performed following manufacturer’s protocol. Data were analyzed using STAR (mm9, (46)), Cufflinks (47), and DESeq (48) using default parameters through Basepair (www.basepairtech.com). Genes altered on chromosome 6 are provided in Table S1.

### Single-cell analysis

Single-cell analysis was performed using the Fluidigm C1 and BiomarkHD system following manufacturer’s protocol. Data were analyzed first using the Fluidigm Real-Time PCR Analysis software to remove any data point with a poor melt curve or no amplification and were further analyzed using R. Cts were normalized to *ACTB* measurements and any cell with an *ACTB* measurement above 8 was excluded as the quality of those samples could not be ensured (Table S2). Data are represented as a difference from the Limit of Detection (as described by Fluidigm, SINGuLAR Analysis Toolset) and expressed as Log_2_Expression. Statistical difference was tested using a Mann–Whitney test with a *p*-value of 0.001. Bimodal distributions were analyzed by calculating the bimodal coefficient (30).

### CUT&Tag

A total of 100,000 cells were collected and processed through the method described in Kaya-Okur *et al.*, 2019, for Total RNA Polymerase II and RNA Polymerase II Ser5P (Cell Signaling Technologies, #54020). Libraries were quantified using the KAPA Quant Kit (#07960140001) and Agilent Tapestation and were sequenced on a NextSeq 500(36 × 36 PE). Data were processed as described in Kaya-Okur *et al*., 2019.

### ATAC-Seq

ATAC-Seq libraries were generated as described previously on cells with the −5 SE floxed and cells with the −5 SE deleted, with Nanog expressed exogenously (41). ESCs were plated 24 h prior to the experiment, collected, and transposed for 30 min. Data were analyzed using bowtie2 (49) using default parameters through Basepair (www.basepairtech.com). Differential peaks were identified using DiffBind (50).

### Data set reanalyses

All Chromatin Immunoprecipitation Sequencing (ChIP-Seq) and Global Run-on Sequencing (GRO-Seq) data sets were displayed using the Integrated Genome Viewer (data.broadinstitute.org). These data sets were previously downloaded and analyzed from the GEO omnibus (13). Data sets are listed in Table S5.

### Statistical analyses

Statistical analyses were done using Microsoft Excel and R. Statistical details of experiments can be found in the figure legends. Two sample two-tailed Student’s *t* test comparisons were performed and *p*-values < 0.05 were considered significantly different. Statistical significance was not shown for values within 20% of the control or between experimental values for RT-qPCR experiments as that is within the error of the assay. All error bars shown in figures are standard deviation between independent experimental replicates. For single-cell RT-qPCR, the Mann–Whitney test was performed and *p* values <0.001 were considered significantly different. Error bars are shown as SD.

## Data availability

All NGS-derived data are available on the GEO (GSE143993). All other data are available upon request to Dr Sridhar Rao (sridhar.rao@versiti.org).

## Conflicts of interest

The authors declare that they have no conflicts of interest with the contents of this article.
