# Supplementary material for: Genome editing demonstrates that the −5 kb Nanog enhancer regulates Nanog expression by modulating RNAPII initiation and/or recruitment
Source: J Biol Chem. 2020 Dec 20;296:100189. doi: 10.1074/jbc.RA120.015152 (PMC7948488; doi:10.1074/jbc.RA120.015152)
Supplement: Table S5 [file mmc6.pdf]

Supporting Figure 1

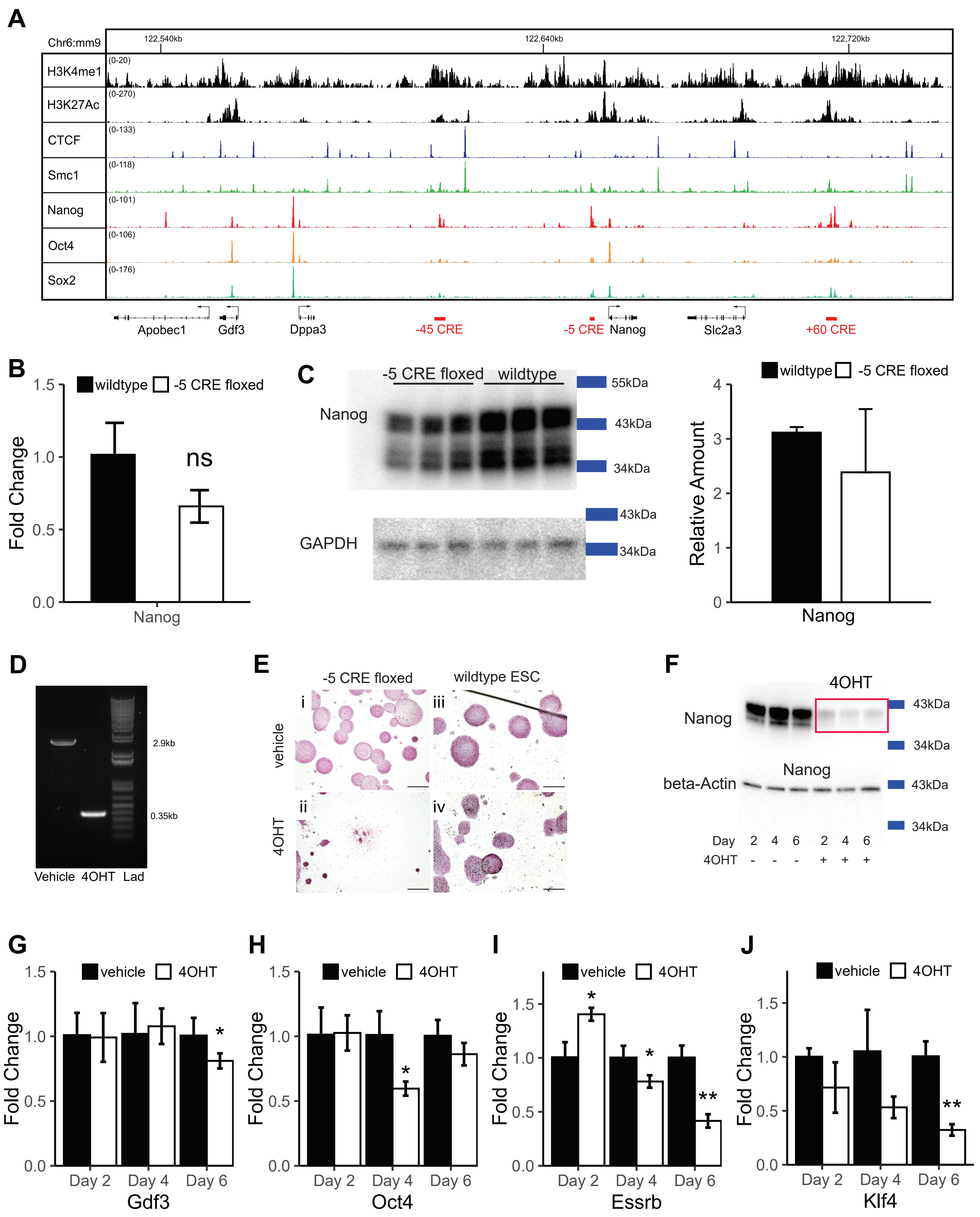

Supporting Figure 1. Nanog and the deletion of the -5 CRE

A. IGV snapshot showing ChIP-seq tracks for H3K4me1, H3K27Ac, CTCF, Smc1, Nanog, Oct4 and Sox2. Genes and CREs are shown below the ChIP-seq tracks. X-axis is genomic position, Y-axis is normalized tag count. **B.** qPCR analysis of *Nanog* expression in wildtype cells versus cells where the -5 CRE is floxed but present. n = 3 **C.** Western blot for Nanog in -5 CRE floxed cells and wildtype cells for three biological replicates per cell line. GAPDH is shown as loading control. Quantification is shown on the right. **D.** PCR genotyping before and after tamoxifen (4OHT) treatment of cells depicted in Fig. 1A **E**. Alkaline Phosphatase of -5 CRE floxed cells (i-ii) and wildtype cells (iii-iv) treated with tamoxifen (4OHT) for six days. Scale bar = 0.5mm. **F.** Western blot for Nanog in -5 CRE floxed cells at different days post 4OHT treatment. Actin is shown as a loading control. Expression for **G.** Gdf3 and **H - J.** pluripotency markers for bulk -5 CRE floxed cells treated with 4OHT or vehicle for 6 days. n = 3 All mRNA levels measured by RT-qPCR. *$p<0.05$, **$p<0.01$, ***$p<0.001$ Student’s Two Sample t-test.

Supporting Figure 2

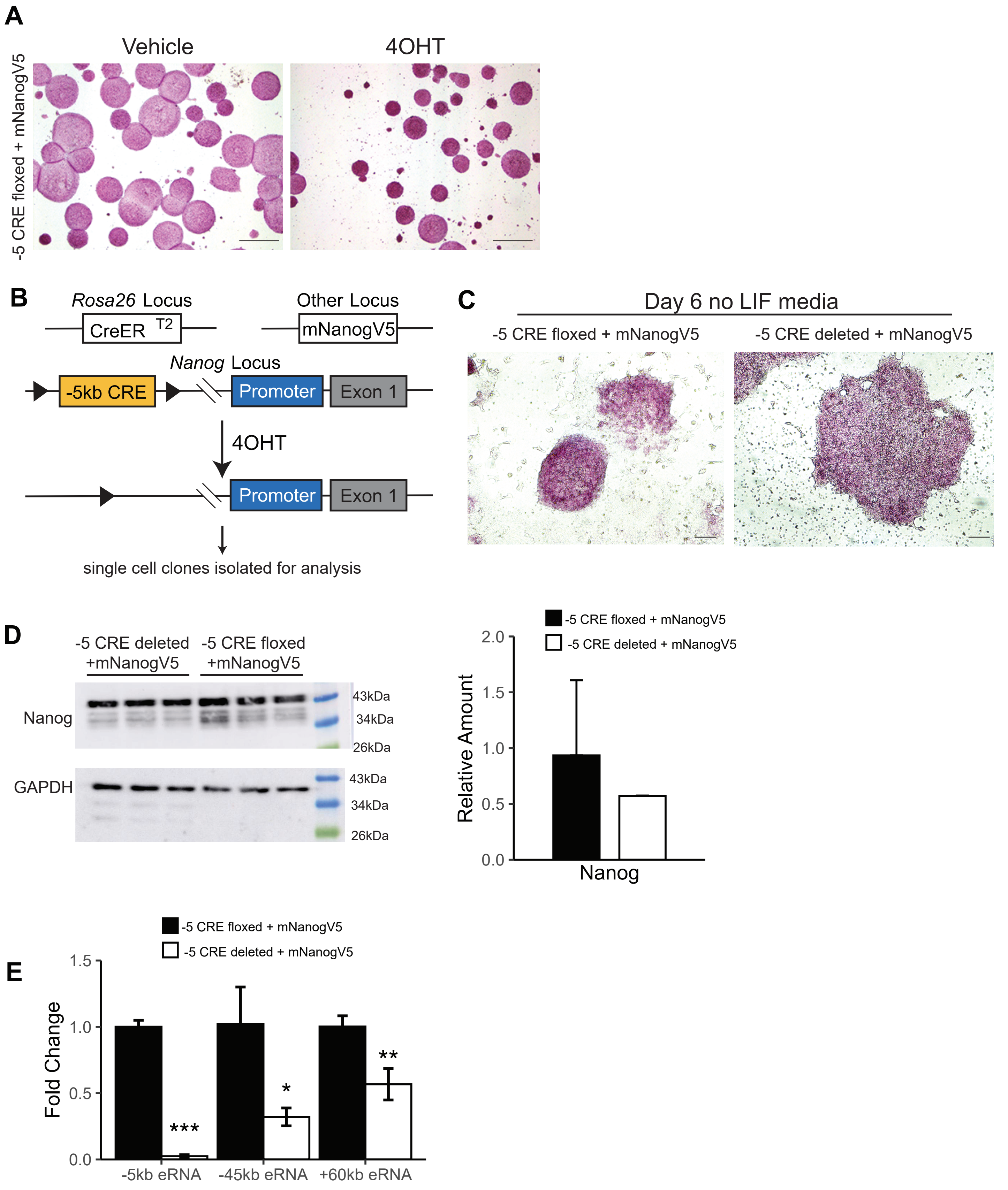

Supporting Figure 2. Stable deletion of the -5 CRE

A. Alkaline Phosphatase staining of -5 CRE floxed + mNanogV5 cells treated with vehicle or tamoxifen (4OHT) for 6 days. Scale bar = 0.5mm **B.** Stable biallelic deletion of the -5 CRE was achieved by rescuing with a mouse Nanog cDNA. Single cell clone were isolated for analysis and used in the rest of the panels in this figure. **C.** Alkaline phosphatase staining of -5 CRE present and deleted cell lines with mNanogV5 expression after 6 days of LIF withdrawal. Scale bar = 0.5mm **D.** Western blot for Nanog in -5 CRE floxed cells or -5 CRE stably deleted cells, both expressing mNanogV5, for three biological replicates per cell line. GAPDH is shown as loading control. Quantification is shown on the right. **E.** Expression of eRNA for the three *Nanog*-associated super-enhancers. n = 3. eRNA levels measured by RT-qPCR. *p<0.05, **p<0.01, ***p<0.001 Student's Two Sample t-test. The presence of a *Nanog* cDNA expressed in *trans* is indicated on the figure as +mNanogv5.

Supporting Figure 3

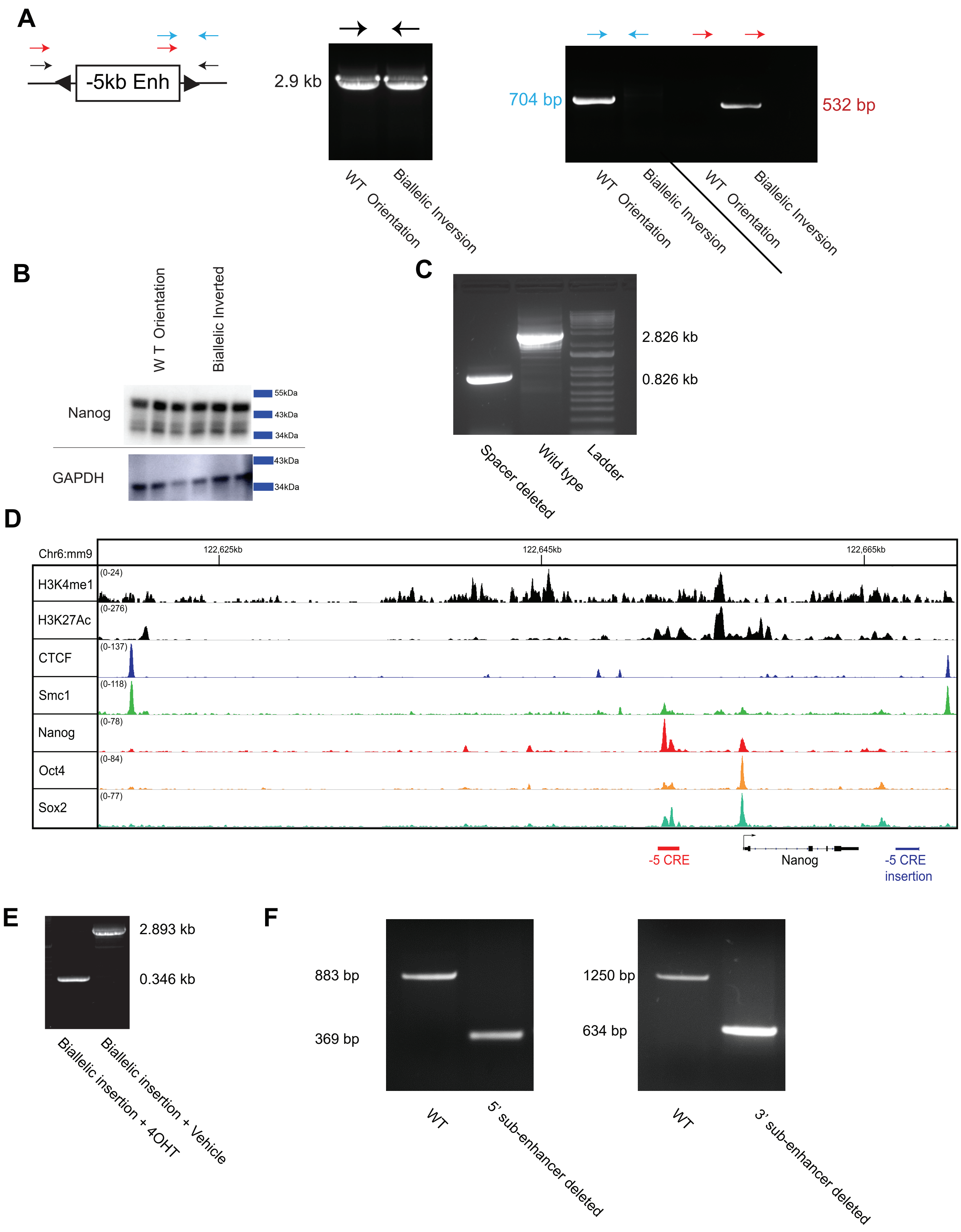

Supporting Figure 3. Manipulation of the -5 CRE

A. Schematic of PCR genotyping strategy of -5 CRE inversion cells shown in Figure 3a (left), with DNA PCR gels shown on right. **B.** Western blot of Nanog in floxed and inverted -5 CRE cell lines for three biological replicates per cell line. GAPDH is shown as a loading control. **C.** PCR genotyping of cell lines where the -5 CRE is closer to the *Nanog* promoter, as shown in Figure 3b. **D.** IGV snapshot of -5 CRE downstream insertion. X-axis is genomic position, Y-axis is normalized tag count. **E.** PCR genotyping for biallelic inserted cells before and after 4OHT treatment. (F) PCR genotyping of the two constituent enhancer deletions. PCR genotyping is labelled with band sizes in kb or bp.

Supporting Figure 4

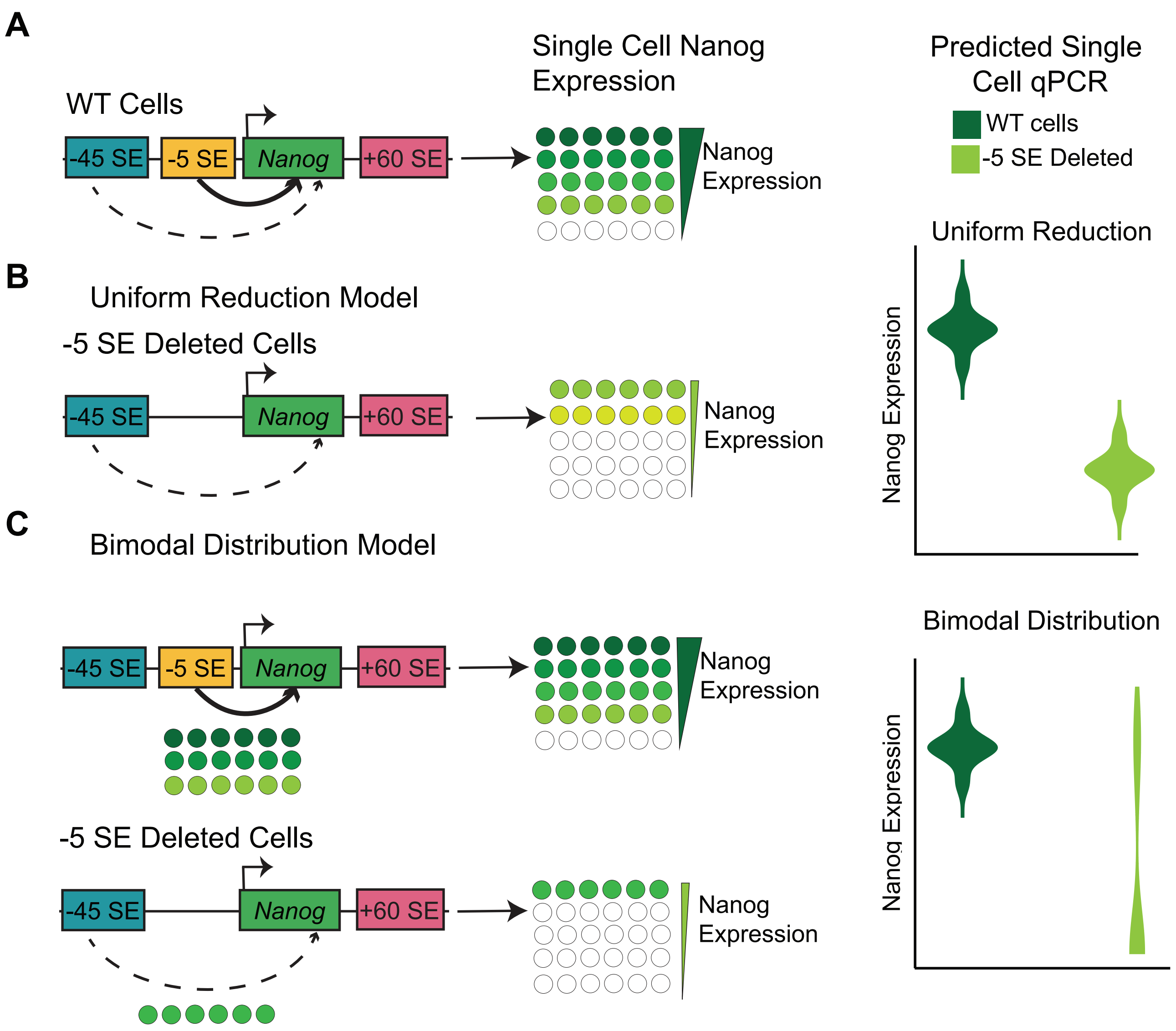

D

	Total Nanog		Nanog 3'UTR	
type	wildtype	deleted	wildtype	deleted
mean	24.526327	23.718673	24.245439	21.639222
standard deviation	0.4190097	0.7454626	0.4523558	0.9061163
skewness	-0.86346	0.5429556	-0.1301826	0.0082883
kurtosis	5.5013898	3.7309816	2.8670937	2.3761335
bimodal coefficient (>5/9 implies bimodal distribution)	0.3172948	0.3470403	0.3546963	0.4208807

Supporting Figure 4. Predicted results of single-cell RT-qPCR based upon different models.
A. Wild-type cells and interaction between *Nanog* and the -45 SE and -5 SE, with the predicted distribution of *Nanog* expression across cells. **B**. In the universal reduction model there is a reduction in *Nanog* expression in all cells following enhancer deletion, implying the -5 SE is active in all cells. **C.** In the bimodal distribution model, the -5 SE regulates a population of cells which have a higher level of *Nanog* expression cells while other enhancers are regulating cells that have lower *Nanog* expression. Upon deletion of the -5 SE, the only cells that remain are ones that regulated by other enhancers that express lower levels of *Nanog*. **D.** Calculations for bimodal coefficient analysis for Total Nanog and Nanog 3' UTR, with the four moments (mean, standard deviation, skewness, and kurtosis).

Supporting Figure 5

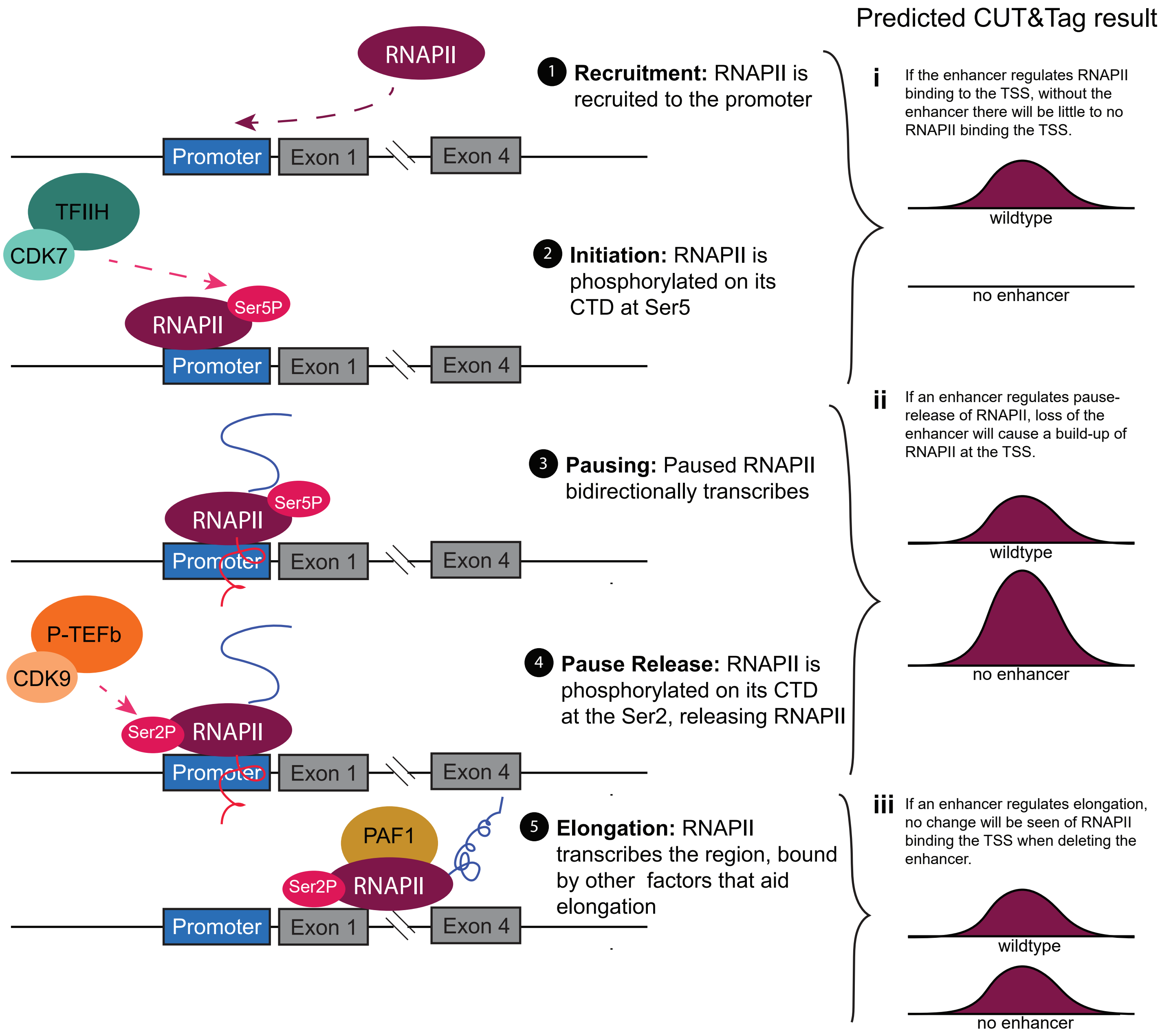

Supporting Figure 5. Enhancer-mediated gene expression

Overview of steps of transcription (1-5) and the predicted changes in Total RNAPII and RNAPII-Ser5P if an enhancer is regulating that step and is deleted (i-iii).

Supporting Table 1. Altered genes in the Nanog TAD and the TADs upstream and downstream from Figure 2 RNA-seq.

Supporting Table 2. Data from single cell experiments for all genes tested.

Supporting Table 3. Cell lines generated in this paper.

Supporting Table 4. 1) gRNAs for generating cell lines. 2) PCR genotyping primers for all cell lines generated in this paper. 3) primers for RT-qPCR.

Supporting Table 5. Accession numbers for published ChIP-Seq datasets analyzed.
